# Cathodic hydrogen production by simultaneous oxidation of methyl red and 2,4-dichlorophenoxyacetate in aqueous solutions using PbO_2_, Sb-doped SnO_2_ and Si/BDD anodes. Part 2: hydrogen production

**DOI:** 10.1039/d0ra03954c

**Published:** 2020-10-21

**Authors:** José Eudes L. Santos, Djalma R. da Silva, Carlos A. Martínez-Huitle, Elisama Vieira dos Santos, Marco A. Quiroz

**Affiliations:** Universidade Federal do Rio Grande do Norte, Instituto de Química Campus Universitário 3000, Lagoa Nova CEP 59078970 Natal RN Brazil carlosmh@quimica.ufrn.br marco.quiroz@ccet.ufrn.br

## Abstract

In this work, results concerning hydrogen gas production during the oxidation of methyl red (MR) and sodium 2,4-dichlorophenoxyacetate (2,4-DNa), is presented, emphasizing not only the amount of hydrogen gas that was produced but also the kinetic and efficiency parameters involved in this process. For this purpose, a two-compartment electrochemical cell was used with a Nafion® membrane as separator in order to collect H_2_ without other chemical species (only with traces of water vapor). Under these experimental conditions, it was possible to guarantee the purity of the H_2_ collected. The electrochemical oxidation of MR and 2,4-DNa solutions was carried out by applying 30 mA cm^−2^ at 298 K, using different non-active anodes (Si/BDD, Pb/PbO_2_, or Sb-doped SnO_2_) and different cathodes (Pt mesh, 316-type stainless-steel, or Pt–10%Rh) in order to investigate the effect of the electrocatalytic materials and experimental conditions. Thus, the H_2_ produced was measured as a function of the electrolysis time and compared with the values estimated by Faraday's law. The results showed that the hydrogen production rate *r*(H_2_) is independent of the nature of the anodic material, although an important effect on the oxygen production was observed on the BDD anode by using sulfuric acid as supporting electrolyte. The effect was discussed through the formation of sulphate-oxidizing species (SO_4_^−^˙ and S_2_O_8_^2−^) which interfere in the oxygen production step on BDD anodes. The use of different cathodes showed small changes in the hydrogen production rate *r*(H_2_), which were basically associated with the differences in hydrogen adsorption energy prior to its evolution. The results were discussed in light of the existing literature.

## Abstract 2

Efficient production of H_2_ from the electrochemical oxidation of organic compounds with non-active anodes in a divided cell.

## Graphical abstract

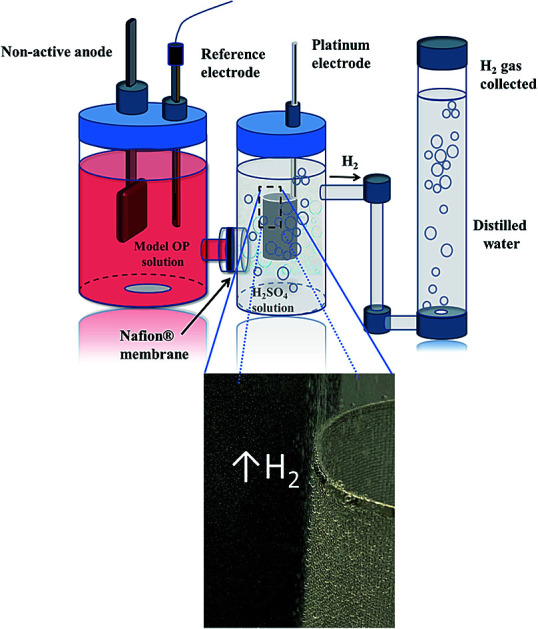

## Introduction

Hydrogen production is now a very important process in various fields of science and technology. Its importance lies in the prospect of becoming a significant option in the search for alternative sources of energy, above all sustainable and compatible with the environment. Hydrogen is not only the essential fuel for the fuel cell technologies but it also serves as an energy carrier^1^ as well as a feedstock for other industries,^2^ including biofuels generation.^3^ This fact is important if the energetic content of H_2_ is compared with the traditional fuels such as gasoline, methane or methanol^4^ based on Lower Hydrogen Value and 1 atm, 25 °C for gases: H_2(g)_ = 33.33 kW h kg^−1^; CH_4(g)_ = 11.39 kW h kg^−1^; CH_3_OH_(l)_ = 5.47 kW h kg^−1^; and gasoline_(l)_ ∼ 12 kW h kg^−1^. This is clearly a great feature to consider, but not only that, H_2_ can be also obtained directly from water, and this is certainly abundant. Thus, the research direction is clear, to find acceptable conditions to make hydrogen production, a profitable process.

The hydrogen production by water electrolysis (as hydrogen evolution reaction, HER) is a well-known electrochemical approach and it has been studied in depth since the mid-twentieth century, especially on cathodes of noble metals (platinum-group metals, PGM) and/or Pt-alloys.^5,6^ Based on the existing literature,^7^ Pt (particularly, in its polycrystalline form) and some of their alloys (such as Pt–Rh) and other metallic substrates (such as stainless steel (SS)) act well as cathodes in acidic media, although SS tends to exhibit certain corrosion level at long electrolysis times. For alkaline medium, Ni-based cathodes seem to be the best option.^8,9^

At standard conditions of pressure and temperature (SPT: 1 bar and 298 K), water splitting requires 1.23 V/RHE or its equivalent in free energy (237 kJ mol^−1^), which must be covered by a suitable electrochemical array. This potential condition is the same regardless of the pH of the electrolyte, and the only difference between the electrolytic medium is the displacement of the electrode potentials which depends on the pH.

Chemical reactions taking place in acidic media (*e.g.*, 0.5 mol L^−1^ H_2_SO_4_) are as follow:

Anode (+):1
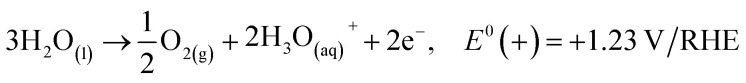


Cathode (−):

2 2H_3_O_(aq)_^+^ + 2e^−^ → H_2(g)_ + 2H_2_O_(l)_, *E*^0^(−) = 0.00 V/RHE

Total:2H_2_O_(g)_ → O_2(g)_ + 2H_2(g)_, *E*_cel_ = +1.23 V/RHE

Chemical reactions taking place in alkaline media (*e.g.*, 1.0 mol L^−1^ KOH) are as follow:

Anode (+):3
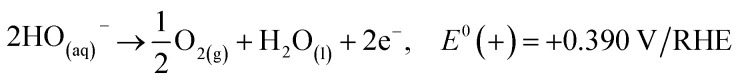


Cathode (−):

4 2H_2_O_(l)_ + 2e^−^ → H_2(g)_ + 2HO_(aq)_^−^, *E*^0^(−) = −0.840 V/RHE

Total:
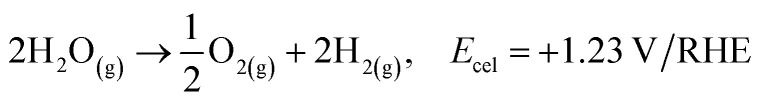


For this reason, the pH-solution is mainly chosen in terms of the electrode material stability, this is, in acidic media the metals corrode, but in alkaline media, passivation is attained. Theoretically, 1.23 V/RHE should be necessary to break the water molecule but the process is kinetically so slow that it has no greater utility. Therefore, it is required to apply a higher voltage to overcome the various barriers that limit the reaction. This voltage requirement depends on various factors such as quality of used materials, temperature, pressure and resistivity of the solution,^10^ for this reason, the industrial electrolyzer-cells are typically operated at temperatures between 70 °C and 90 °C by applying from 1.8 to 2.2 V/RHE.^11^ Owing to these operating conditions, only an energy percentage between 56–73% is storage in a form of hydrogen gas.^12^ Commercially, the alkaline electrolyzer-cells with diaphragm as separators and those with polymer electrolyte membrane (PEM) are the water electrolysis technologies with the best cost/benefice relationship. However, the PEM systems has the advantage to produce hydrogen gas with a high level of purity (99.99%) due to the low gaseous permeability of the membrane.^13^

Recently, more options have shown that photoelectrochemical cells can be also used to produce H_2_ by using materials photosensitive to UV and/or visible radiation as electrodes.^14,15^ The essential concept behind this technology is to use UV-vis radiation on a semiconductor material to generate sufficient energy to provoke the break of the water molecule, extracting electrons and forming H_2_ and O_2_. This approach is known as water photoelectrolysis. This method has an important advantage over the traditional electrolysis because the overpotential required to the water splitting on an illuminated semiconductor is lower than on a metallic material, which diminishes the energetic costs and increases the percentage of electrical current converted to hydrogen.^16^ In this case, the water decomposition in an acidic media, for instance, is carried out through the hole generated in the semiconductor electrode while the electrons are used in the counter electrode to produce hydrogen gas,^16^

5 4H_(ac)_^+^ + 4e_(aux)_^−^ → 2H_2(g)_

6 2H_2_O_(l)_ + 4h_(sc)_^+^ → 4H_(ac)_^+^ + O_2(g)_

Moreover, since the holes can produce hydroxyl radicals, then it is possible that the following steps also take place,^17^

7 ˙OH + ˙OH → H_2_O_2_

8 H_2_O_2_ + 2h^+^ → O_2(g)_ + 2H^+^

Since the oxidation potential value of the hydrogen peroxide (−0.695 V/RHE)^18^ is lower than that of the water electrolysis (1.23 V/RHE), this last step is always carried out in the photoelectrolysis cells designed to water splitting.^17^ Several semiconductor materials have been studied for this purpose,^19–21^ but since the Fujishima–Honda work in 1972 (ref. 22) until now; the n-type titanium dioxide (TiO_2_) with and without modifications has been the semiconductor-based electrode more investigated as photocatalysts for hydrogen production.^23–29^ In these cases, the amount of hydrogen gas produced (in mL or μmol units) by photoelectrolysis is very variable so, for instance, in the work of Lee *et al.*^25^ a production of 10.2 mL after 9 h on a 0.1 mol% Zn–TiO_2_NTs photocatalyst was attained; ∼2.34 mL h^−1^ cm^−2^ on Gd^3+^:TiO_2_ as reported by Sudhagar *et al.*,^27^ or 8.53 μmol g_cat_^−1^ as reported by Simamora *et al.*^26^ These photoelectrochemical approaches produce H_2_ with relative good photoconversion efficiency, but with a low production level yet.

From an economic point of view, it is necessary to design electrolytic devices that make water electrolysis a productive and profitable process. In this sense, alternatives directly associated with the cathodic production of hydrogen are being investigated, such as the use of sacrificial organic compounds^30^ in the anodic semi-cell or the oxidation of organic pollutants^31^ as a part of electrochemical cells for wastewater treatment. Recently, it was proposed the electrochemical oxidation of methanol as a sacrificial compound to produce hydrogen gas^30^ in a proton exchange membrane electrolysis cell (PEMEC). The methanol oxidation on a Pt–Ru (1 : 1)/C anode allowed to diminish significantly the energetic requirements for water electrolysis, achieving efficient hydrogen production (14.5 cm^3^ at 100 mA after 20 min of electrolysis of a 2 M methanol solution). This process was only dependent on the applied current (strictly obeying the Faraday's law); and the possible deactivation of the anodic electrocatalyst was considered due to the CO formation from methanol oxidation. As mentioned by Lamy *et al.*,^30^ the use of a sacrificial analyte seems to be a good alternative for the water splitting to produce H_2_ because it reduces the amount of energy expended in the electrolysis process. Another option, close to the previous one, is to consider the H_2_ production as the subsequent stage of a primary step of greater interest, such as the case of the electrooxidation of organic pollutants.^31,32^ In this sense, the oxidation remains as the target of the electrochemical treatment process, but now the cathodic process takes a more effective attention.^33^ However, the way in which the production of H_2_ is considered is not consistent with the purpose of obtaining it clean yet, in attractive quantities and at low energy expenditure. For example, in the work of Jiang *et al.*^31^ H_2_ was produced at a rate of ∼1400 mL h^−1^ using a BDD anode to oxidizer 4-nitrophenol and by using stainless as cathode to produce H_2_, although the current density used seems to be high (∼250 mA cm^−2^), no enough information about the H_2_ production was given. In that work as in others, the H_2_ production was performed in one compartment electrochemical cells, assuming that the complete mineralization of the organics was achieved; however, the amount of hydrogen produced is only a fraction of the total of gas that could be generated (as will be explained here). The separation of gases, essentially hydrogen, oxygen, but also carbon oxides, volatile intermediates and others, makes the use of single cells unattractive; therefore, the design of double compartment cells is part of the study of H_2_ production with oxidation of organic compounds, as the associated stage.^30,31,33,34^ Therefore, the aim of this second part of this exhaustive experimental work, it was not to study the HER itself, but so the capacity of the electrochemical oxidation system to promote, as combined approach, the production of reasonable quantities of clean H_2_ in the cathodic half-cell. For this purpose, a two compartment PEM cell was used under galvanostatic conditions (30 mA cm^−2^) according to the established in the first part of the work.^35^ Preliminary, electrochemical behaviors and considerations were previously obtained and discussed respectively, regarding the cathodic material. The hydrogen volume was determined and compared with the theoretical estimations using Pt cathode. Oxygen evolution was also considered in order to comprehend as this secondary reaction, during electrochemical oxidation of organics, could affect the production of hydrogen. Faradaic efficiencies were then determined to establish to determine the optimal operating conditions.

## Experimental section

Chemicals and electrode materials used as anodes were described in the first part of this work. For electrochemical oxidation were chosen the methyl red (MR) dye and the sodium 2,4-dichlorophenoxyacetate (2,4-DNa) (puriss. p.a. ≥ 99.5%) herbicide as model organic pollutants (OP) in aqueous acidic medium in order to favor the solubility conditions. As anodes were used, Pb/PbO_2_, Ti/Sb-doped SnO_2_ and Si/BDD electrodes (considered as non-active anodes); while as cathodes were used, Pt and Pt–10%Rh wires as well as a 316-type stainless-steel (SS-316) rod. All electrodes were used in 0.25 mol L^−1^ Na_2_SO_4_ solution as supporting electrolyte, and an Ag/AgCl (KCl 3 mol L^−1^) as the reference electrode.

**Fig. 1:**
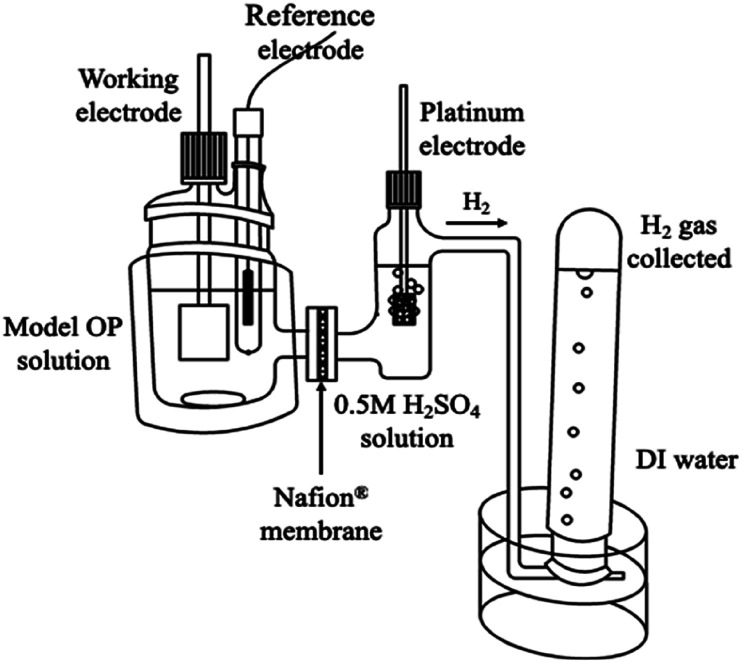
Electrochemical cell PEM type used to study the electrochemical oxidation of OP (anodic reservoir) and the simultaneous HER (cathodic compartment). Hydrogen gas is collected over DI water in an inverted burette. Array at 298 K and 1 atm.

The electrochemical experiments were developed in a two-compartment cell, both separated by a Nafion® membrane of 350 and/or 417 type with the opaque face towards catholyte solution (PEM cell). Hydrogen gas was collected in deionized (DI) water and it was measured by using an inverted burette directly connected to the cathodic compartment which has a capacity of 100 mL, whereas the anodic compartment having a capacity of 250 mL, Fig. 1.

The experimental conditions were those of the electrochemical oxidation by applying 30 mA cm^−2^ at 25 °C and under stirring condition (∼350 rpm). The hydrogen gas produced at the cathodic compartment where it was collected in an inverted burette with defined temperature and pressure conditions in order to take into account the vapor pressure of water to calculate the actual volume of H_2_ produced. It was measured at the same time intervals used to monitor the electrochemical oxidation of the OP.

## Results and discussion

### About the electrochemical oxidation of MR and 2,4-DNa

**Fig. 2:**
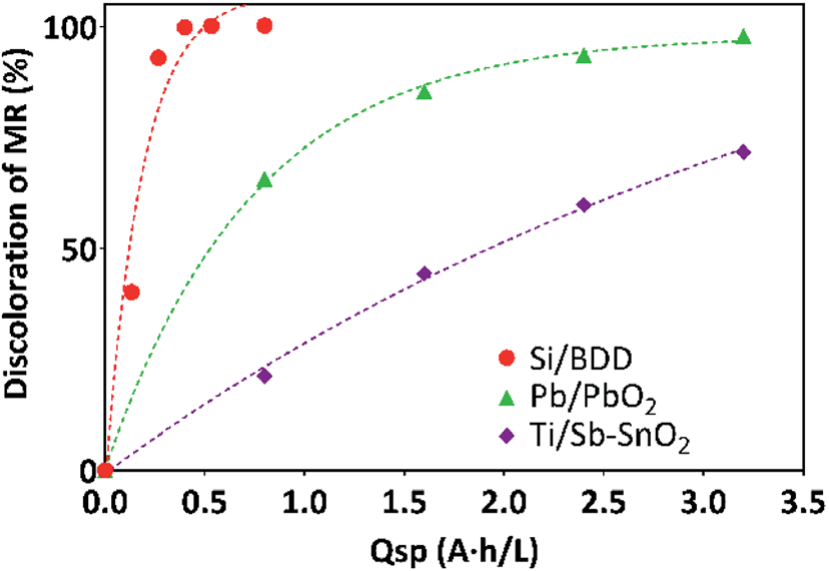
Electrochemical oxidation of 0.5 mol L^−1^ H_2_SO_4_ + 20 ppm MR solutions (as % of discoloration) at 30 mA cm^−2^ and 298 K as a function of the electrolysis time.

In a previous work,^35^ we have shown that PbO_2_, Sb-doped SnO_2_ and BDD anodes are efficient electrocatalytic materials to oxidize OP such as the MR and 2,4-DNa, which are considered as target compounds in various wastewater treatments.^32^ The oxidation taken place *via* the action of hydroxyl radicals (˙OH) formed by water discharge on the anode surface, as well as *via* the participation of sulphate-oxidizing species (SO_4_^−^˙ and S_2_O_8_^2−^), when BDD anode was used.^36^ It was observed that the MR oxidation was less complex than that attained by 2,4-DNa, considering 60 min of electrolysis time as sufficient to complete its elimination, Fig. 2.

The electrocatalytic activity (*a*) values estimated from the corresponding figures of the apparent rate constants derived from the kinetic analysis of the oxidation curves (*k* in units of min^−1^) were: 13.8 × 10^−8^, 4.4 × 10^−8^ and 2 × 10^−8^ mol min^−1^ cm^−2^ for BDD, PbO_2_ and SnO_2_ anodes, respectively. This sequence in electrocatalytic activity is consistent with the increase on the overpotential for the oxygen evolution reaction (OER) from BDD to Sn-doped SnO_2_ anodes.

Meanwhile, the electrochemical oxidation of 2,4-DNa was a more complex process due to the presence of chlorine groups directly bonded to the aromatic ring of the 2,4-DNa molecule. For this case, Tafel analysis showed that chemical and/or electrochemical processes taken place involving electron transfer processes in some particular sequence, being more evident when 2,4-DNa was present in the electrolytic solution. This fact highlights the participation of both type of oxidizing species (˙OH as well as SO_4_^−^˙ and S_2_O_8_^2−^),^37^ but with the formation of the 2,4-dichlorophenol (2,4-DP) intermediate as first oxidation step. This formation of this intermediate was mainly induced by the participation of sulphate-oxidizing species (SO_4_^−^˙ and S_2_O_8_^2−^). It is important to point out that the oxidation of 2,4-DNa was completed at electrolysis times longer than 60 min due to the complexity nature of the molecule.

The electrochemical oxidation results showed, in general terms, that, all non-active anodes had a great capacity to produce efficiently ˙OH.^35^ It was established that ˙OH are necessary to the formation of sulphate-oxidizing species (SO_4_^−^˙ and S_2_O_8_^2−^)^38^ as well as each one of the oxidizing species can participate, directly, indirectly, or collaboratively to influence the OER or to intervene in the oxidation of the organic pollutants.^32^ These last behaviors are more evident when H_2_ production is analyzed in the current second part of this study.

### Polarization curves for the HER on Pt, Pt–10%Rh and SS-316

In order to determine the electrochemical behavior at the potential at which the HER start, on the cathodic materials used, the corresponding polarization curves (PC) were obtained by using a three electrodes system: a Pt mesh as counter-electrode and an Ag/AgCl (3 mol L^−1^ KCl) as reference electrode; nevertheless, the potentials were quoted respect to the normal hydrogen electrode (RHE) for which the follow conversion equation was used:

9 *E*(RHE) = *E*(V/(Ag/AgCl, 3 mol L^−1^ KCl)) + 0.1942 + 0.0591pH

**Fig. 3:**
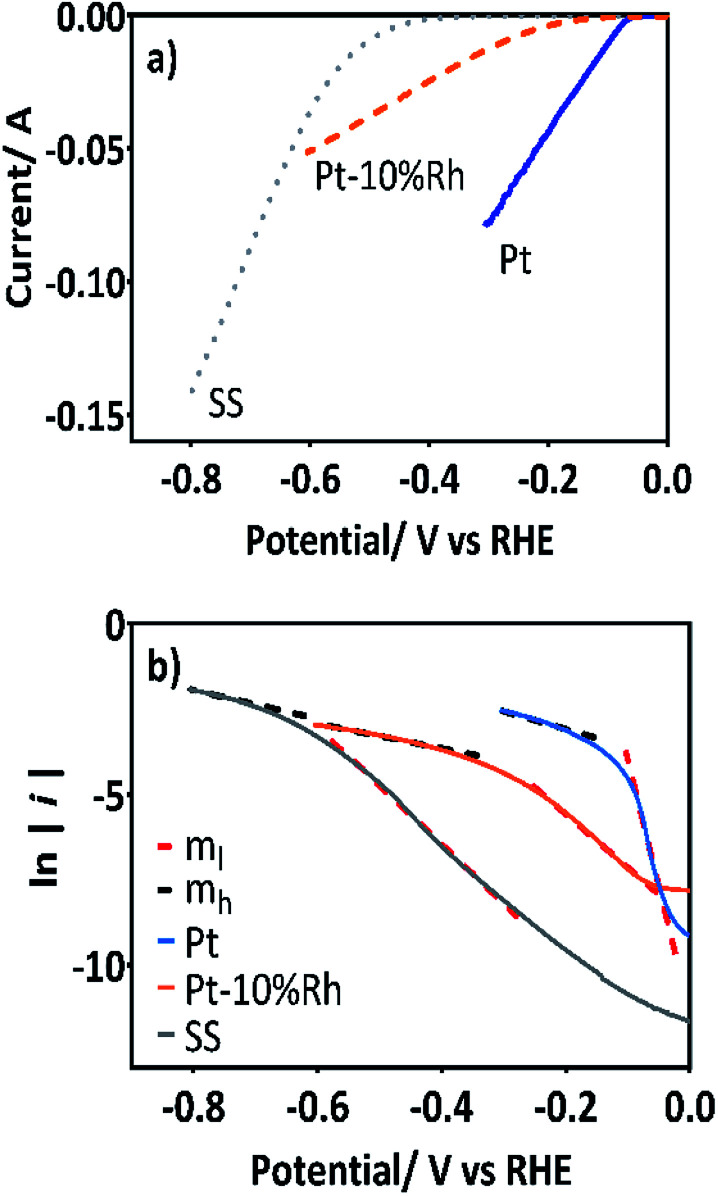
(a) PC of the Pt (blue), Pt–10%Rh (orange) and SS (gray) cathodes for HER in 0.5 mol L^−1^ H_2_SO_4_ (LSV scan rate = 5 mV s^−1^). (b) The corresponding Tafel plots of Pt (blue), Pt–10%Rh (orange) and SS (gray) calculated from data of plots in (a). Dashed lines show the overpotential regions at which the Tafel slopes were calculated.

The cathodes were Pt wire, Pt–10%Rh wire in a spiral form over a glass rod and a SS-316. Since the chemical characteristics of the HER are dependent on the pH of the electrolyte, the cathodic PC were obtained in 0.5 mol L^−1^ H_2_SO_4_ solution at 25 °C using a linear sweep voltammetry (LSV) technique. The initial potential was fixed at the corresponding open circuit potential (*E*_*i*=0_) for each one of the cathodes used and swept in cathodic direction until about −0.8 V/RHE at a scan rate of 5 mV s^−1^, Fig. 3a.

As can be observed in Fig. 3a, the Pt and Pt–10%Rh cathodes have the lowest onset overpotential, ∼0.023 and ∼0.065 V/RHE, respectively; while for the SS-316 electrode the onset potential for the HER was extended until ∼0.34 V/RHE. These results are consistent, with the fact, that Pt is the best electrocatalyst for HER,^39^ although Pt–10%Rh (ref. 40) and SS-316 have also good electrocatalytic activity to produce hydrogen in acidic media.^41^ From the PC, the corresponding Tafel lines were plotted with the overpotential in V *vs.* the hydrogen potential. As can be observed in Fig. 3b, two Tafel segments were used for each one of the cathode tested in order to estimate the Tafel slopes (see Table 1), at lower and higher overpotential regions (m_l_ and m_h_, respectively, in Fig. 3b).^42^

**Table 1:** Tafel slopes and potentials for cathodic materials

Cathode	Tafel slope (mV per decade)		H_2_ onset (V/RHE)
	Low *η* region	High *η* region	
Pt	13.1	184	∼−0.023
Pt–10%Rh	59	311	∼−0.065
SS	57.8	203	∼−0.345

These values of Tafel slopes suggest a Volmer–Tafel route for the HER at these cathodic materials in 0.25 mol L^−1^ H_2_SO_4_ solution and 298 K, involving an adsorbed H intermediate (MH_ads_):^42^

Volmer step:

10 H_3_O_(ac)_^+^ + e_(M)_^−^ ↔ M(H_ads_) + H_2_O_(l)_

Tafel step:

11 2M(H_ads_) ↔ 2M + H_2(g)_

in accordance with the kinetic model for the HER on polycrystalline surfaces described by Gennero de Chialvo^43^ on the basis of a mathematical simulation of the Volmer–Heyrovsky–Tafel mechanism. For the Tafel slope values higher than 120 mV per decade, a multiple reaction pathway with surface blockage of adsorbed hydrogen was suggested.^39,44^ Therefore, from the results obtained, the HER is efficiently conducted at cathodic materials selected, under the selected experimental conditions, indicating that, an efficient hydrogen production will take place during the oxidation of organic pollutants. Nevertheless, as indicated by Tafel plots, the Pt material has a lower hydrogen overpotential, which favors an efficient production of hydrogen. For this reason, it was chosen for the next experiments.

### Hydrogen production on Pt cathodes by anodic oxidation of MR and 2,4-DNa at PbO_2_, BDD and Sb-doped SnO_2_ anodes

As previously established, the hydrogen production was simultaneously measured during MR and 2,4-DNa oxidation in a double compartment cell separated using a Nafion®-417 membrane (PEM cell), Fig. 1. The Nafion®-417 membrane increased the ohmic resistance of the solution between the cathode and anode materials, but it prevented the transport of the oxidation products from the solution to the cathodic reservoir, affecting the cathode electrocatalytic properties. In this way, we can ensure that the collected hydrogen is free of contaminants, except for the water vapor that accompanies it and that, later, it can be easily removed with a desiccator.

Since the oxidation of the organic compound (MR and 2,4-DNa) is the target of the complete electrochemical process, the operating conditions of the PEM cell were those of this procedure, that are, by applying 30 mA cm^−2^ in acidic media at 25 °C. The anodic material was PbO_2_ (8 cm^2^), Sb-doped SnO_2_ (6 cm^2^) and BDD (7.5 cm^2^), and a Pt-mesh as cathodic material. In all cases, a 0.25 mol L^−1^ H_2_SO_4_ + 20 ppm MR solution or a 0.5 mol L^−1^ H_2_SO_4_ + 100 ppm 2,4-DNa solution were used as model aqueous effluents for the electrochemical treatment in the anodic compartment, while a solution of 0.25 mol L^−1^ H_2_SO_4_ was employed as catholyte. Under these conditions, the quantity of H_2_ produced will only depend on the electrolysis time according to the equation:^45^12
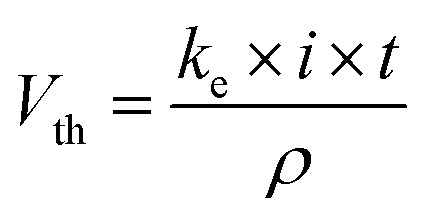
where *k*_e_ = (*M*/*nF*) is the electrochemical equivalent (kg A^−1^ s^−1^), *i* is the applied total current (A), *t* the electrolysis time (s), *ρ* the hydrogen gas density (kg m^−3^), *M* the molar mass of hydrogen (kg mol^−1^), *n* the electron number (2 for H_2_) and *F* the Faraday constant (96 487 C mol^−1^).

**Fig. 4:**
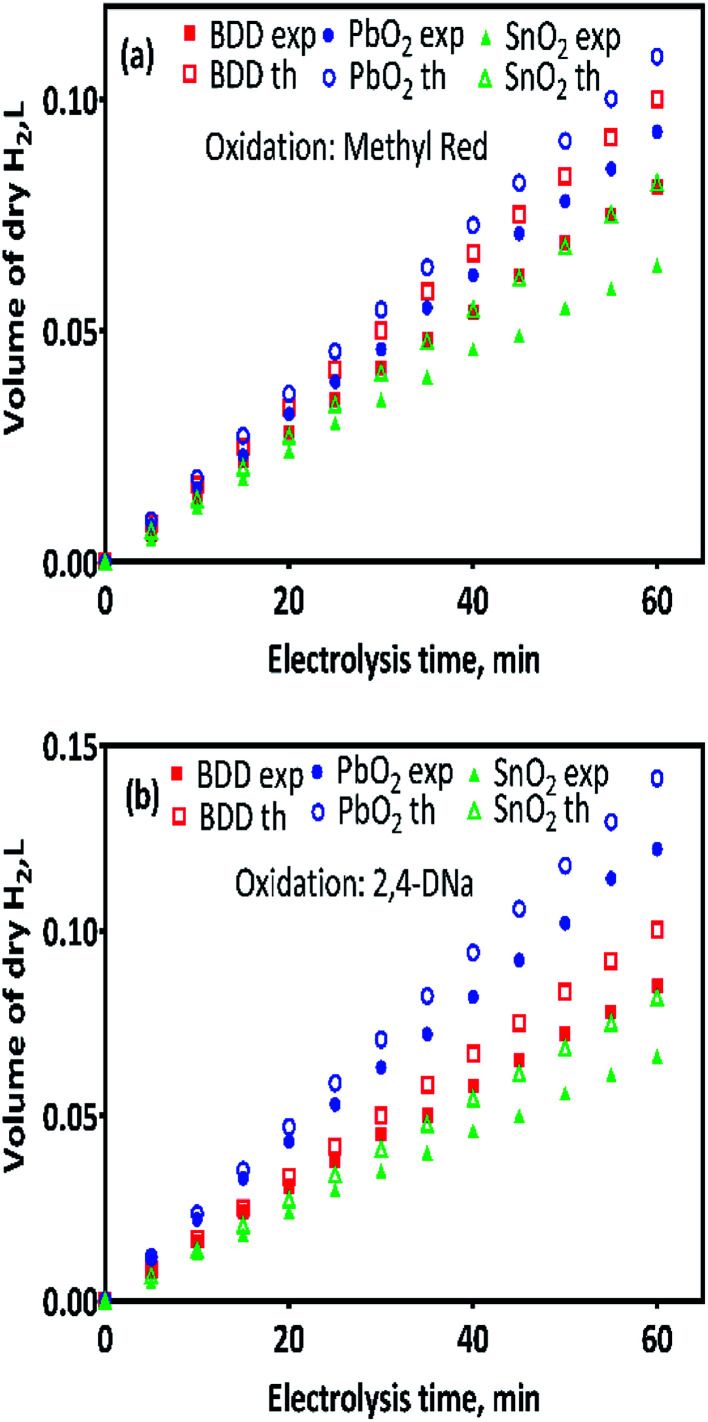
The amount of the hydrogen gas (in L) obtained by theoretical (th) calculation (empty symbols) and by experimental (exp) measurements (full symbols) as a function of the electrolysis time: (a) 0.5 mol L^−1^ H_2_SO_4_ + 20 ppm MR and (b) 0.5 mol L^−1^ H_2_SO_4_ + 100 ppm 2,4-DNa solutions, by applying 30 mA cm^−2^ at 298 K. Hydrogen gas produced at Pt cathode in 0.25 mol L^−1^ H_2_SO_4_ as supporting electrolyte and collected in inverted burette.

Fig. 4a and b show the volume of hydrogen gas produced during the electrochemical oxidation of MR and 2,4-DNa, as a function of the electrolysis time, respectively. To determine the volume of H_2_ produced, a correction was done considering the quantity of water vapor generated in the gas collector due to the hydrogen gas evolution on the cathode. After that, a comparison was done between the experimental and theoretical values achieved, concerning the volume of hydrogen, eqn (12).

As can be observed in Fig. 4, under galvanostatic conditions, the measured volume of hydrogen produced during the oxidation of the organic compounds varied linearly as a function of the electrolysis time, showing in all experimental cases a correlation factor *R*^2^ > 0.995, just as predicted by the eqn (12) and in a good agreement with the Faraday's law. It was also observed, that as electrolysis time increases, the volume difference between the calculated and the experimental values also increases, mainly in the case of the Sb-doped SnO_2_ anodes, even when the change is not greater than 20%. These differences can be associated to a physical effect than that a process effect, in other words, it could be due to the way in which the hydrogen gas is collected. It is important to consider that, the volume of water vapor in the gas collector also increases with the electrolysis time. Furthermore, the existence of intrinsic factors to the process of hydrogen evolution^46^ influence its efficient production, such as the cell design, the electrodes separation, the use of a gas separator, the displacement of gas bubbles from the cathodic surface to the collector and/or parasitic currents.^47^ Therefore, faradaic efficiency (FE), as estimated by the eqn (13), was not 100%; but it is maintained at levels greater than 80%, as can be verified in Fig. 5.13
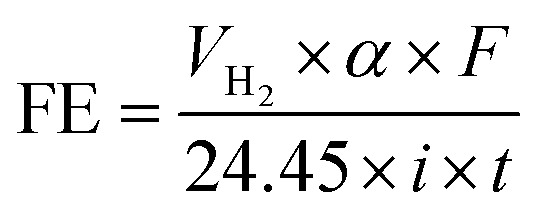
where *V*_H2_ is the volume of hydrogen collected at the time *t*, *α* is the number of electrons transferred (2 for H_2_), *F* is the Faraday constant (96 487 C mol^−1^), 24.45 is the molar volume of a gas at 298.15 K at 1 atm, *i* is the current intensity, and *t* is the electrolysis time.

**Fig. 5:**
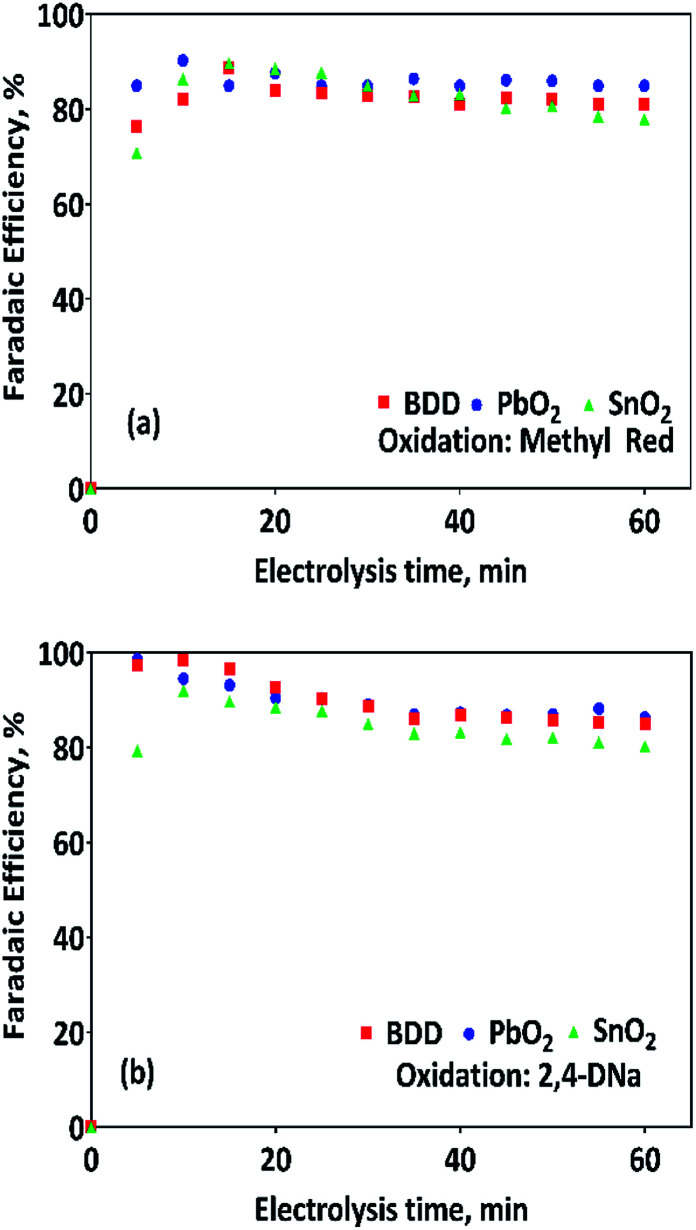
Faradaic efficiencies (in %) calculated by using eqn (13) for (a) data of collected about the volume of hydrogen of Fig. 4a and (b) for that values of collected volume of hydrogen of Fig. 4b.

Although it has been suggested that the hydrogen production is independent of the anode nature;^30^ an indirect effect linked to the overpotential of the oxygen evolution reaction (OER) was attained as well as to the water discharge over the anode,^32^ and consequently, this behavior could affect the hydrogen production rate. The hydrogen production rate, *r*(H_2_), on Pt as cathode and for the anodes used in the electrochemical oxidation of MR and 2,4-DNa, was calculated from the corresponding plots of Fig. 4 and are reported in Table 2.

**Table 2:** Hydrogen production rate on Pt as cathode and for the anodes used in the electrochemical oxidation of MR and 2,4-DNa

Anode	*r*(H_2_), L min^−1^	
	ECOx MR	ECOx 2,4-DNa
Pb/PbO_2_	0.0016	0.0020
Si/BDD	0.0014	0.0014
Ti/Sb-doped SnO_2_	0.0011	0.0011

Jiang *et al.*^31^ showed during the oxidation of 4-nitrophenols that the use of BDD as anode increases the *r*(H_2_) due to its wide potential window. It was assumed that the greater potential difference of BDD, with respect to Pt and Ta, in the potential window, also as anodes, induces a greater flow of electrons from anode to cathode and, consequently, a higher *r*(H_2_) is attained. However, no significant changes in *r*(H_2_) were observed when a comparison between BDD and traditional anodes, such as PbO_2_ and SnO_2_, was done during the oxidation of MR and 2,4-DNa, as shown in Table 2.

**Scheme 1:**
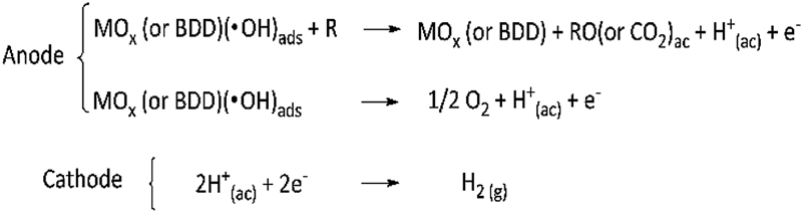
Proposed anodic and cathodic reactions during to electrochemical oxidation of organic pollutants coupled to hydrogen production.

Certainly, a small change in *r*(H_2_) was achieved when different anode was used; nevertheless, this change may be associated to the electrocatalytic properties of the anodic material (those responsible for oxidizing the organic compound) than their wide potential window (which originates the oxygen overpotential), especially at galvanostatic conditions. Thus, it is the electrooxidation of the organic compound and the oxygen evolution that originate the flow of protons toward to the cathode, through the PEM separator, to produce the hydrogen gas,^48^ as well as the applied current which established the potential difference that drives the electrons required to complete the formation of the molecular hydrogen, Scheme 1.

### Oxygen production at PbO_2_, BDD and Sb-doped SnO_2_ anodes

**Fig. 6:**
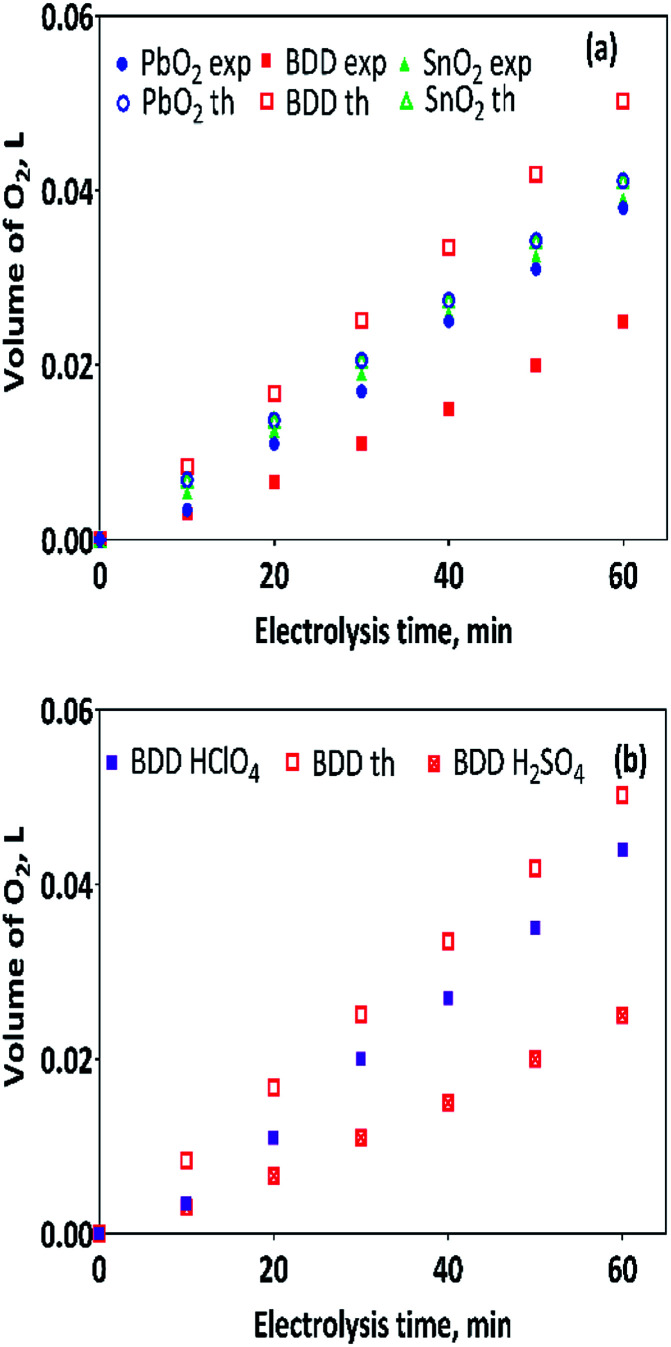
The amount of the gas (in L) obtained by theoretical calculation (empty symbols) and by experimental measurements (full symbols) as a function of the time for: (a) 0.5 mol L^−1^ H_2_SO_4_ + 20 ppm MR and (b) 0.5 mol L^−1^ H_2_SO_4_ + 20 ppm MR (red symbol) and 0.5 M HClO_4_ + 20 ppm MR (violet symbol) solutions at 30 mA cm^−2^ and 298 K.

As discussed in the previous section, the protons required for hydrogen production are generated by the electrocatalytic reaction as well as the oxygen evolution, both of them occurring simultaneously at the anode. This behavior could, in a first instance, explain the results of *r*(H_2_) on the cathode of Pt. However, it is still not so clear because with BDD a greater amount of H_2_ is not produced since it has the highest overpotential of oxygen evolution. Thus, we believe that this is precisely where the answer lies. When the amount of oxygen produced is compared at each one of the anodes here used, a substantial difference, between BDD electrode and the PbO_2_ and Sb-doped SnO_2_ anodes, is attained. As observed in Fig. 6, the amount of oxygen produced with BDD anode is lower than that produced on the other anodes, but further, is just half of the expected theoretical amount of oxygen, Fig. 6a.

This behavior was only observed when H_2_SO_4_ was used as supporting electrolyte in the anolyte.^35^ Conversely, when HClO_4_ was used as supporting electrolyte; the volume of oxygen produced is similar to those produced at the other anodes, Fig. 6b. In our previous work,^35^ it was proposed that by using H_2_SO_4_ as supporting electrolyte the oxygen production through ˙OH radical physically adsorbed on BDD surface, is limited by the formation of sulphate-oxidizing species (SO_4_^−^˙ and S_2_O_8_^2−^),^35,36,38^BDD(˙OH) + SO_4_^2−^ → BDD(SO_4_^−^˙) + OH^−^

Furthermore, the oxygen production rate *r*(O_2_) at BDD anode in H_2_SO_4_ (0.0004 L min^−1^) is also the half-value of the estimated value than in HClO_4_ (0.0008 L min^−1^), therefore, it is reasonable to suppose that the hydrogen production rate should be also minor in H_2_SO_4_, such as is reported in Table 2.

### Hydrogen production on Pt–10%Rh and stainless-steal (SS) rod (316-type) cathodes by anodic oxidation of MR at BDD anode

In the first part of this work,^35^ the electrochemical oxidation of MR and 2,4-DNa was almost completed at BDD anode in 0.25 mol L^−1^ H_2_SO_4_ by applying 30 mA cm^−2^ in electrolysis time not longer than 60 min. Under these conditions, a lower hydrogen production rate at Pt cathode was achieved. Nevertheless, thinking about economic requirements, the use of Pt as a cathode is not practicable; then, Pt–10%Rh or SS-316 were proposed as cheaper cathodic materials, even when these materials have higher hydrogen overpotential (see Fig. 3). In these cases, the hydrogen production rate was slightly increased by using Pt–10%Rh and SS cathodes, 0.0017 and 0.0017 L min^−1^, respectively, respect to the *r*(H_2_) with Pt 0.0014 L min^−1^. This behavior is related to the oxidation conditions where the hydrogen evolution at the Pt–10%Rh and SS-316 cathodes is more stable and less affected by the large release of gas bubbles than in the Pt mesh. These results showed two relevant aspects: (a) the PC evidenced that Pt is the good electrocatalysts for the hydrogen production; (b) at a fixed current density, which was established for the organic-pollutant oxidation, the changes in the hydrogen production rate *r*(H_2_) depends on the nature of the cathodic material due to the differences in the adsorption energy of the hydrogen atoms prior to the evolution of the molecular hydrogen.^49,50^ Therefore, although the SS-316 showed good properties for producing hydrogen, in terms of the amount as well as the rate, this is the cathodic material most vulnerable to corrosion in acidic media.

The possibility of recovering energy from the electrolysis of wastewater by the product of the counter-electrode reaction, the evolution reaction of hydrogen, which is generally ignored, is a viable alternative to recover part of the energy consumed in these processes. Hydrogen gas is obtained at no additional cost since the reduction reaction is the result of the oxidation reaction of the solvent, electrolyte, or organic pollutants in the wastewater. Approaches to recover part of the energy consumed by electrochemical technologies with hydrogen production and use of anodic depolarization that reduces the anodic overpotential, which is the main responsible for the high energy consumption, can recover up to 90% of the incoming energy. The use of renewable energies as hybrid electrolysis systems eliminates the energy cost, making hydrogen production viable.^51,52^

## Conclusions

Nowadays, the hydrogen production is a subject of great scientific and technological interest, among other reasons because this is one of the most important energy alternatives to the use of fossil fuels. However, obtaining it at the best cost, it is still a great challenge, and all possible options should be able to be considered. Hydrogen is the natural product in the oxidation process of aqueous organic pollutants, since this is produced in the complementary cathodic reaction of the net process of electrochemical treatment. At this point, it is relevant to know whether or not; we are interested in collecting the hydrogen produced in the electrochemical cell, since the type of cell that will be used will depend on this decision. In this work, a two-compartment electrochemical cell separated by a Nafion® membrane was used in order to collect the hydrogen produced in the cathodic compartment while the oxidation of the organic compound occurred simultaneously in the anodic compartment under the galvanostatic fixed condition.

Thus, in the electrochemical oxidation of MR and 2,4-DNa in acidic media using PbO_2_, Sb-doped SnO_2_ (MO_*x*_ type electrodes) and BDD anodes, the electrochemical oxidation occurs with a good discoloration level for MR solutions and with a partial degradation of 2,4-DNa into the interval time fixed for the electrolysis. The trends of the results suggested a complete oxidation for both organic pollutants at longer electrolysis times. However, it was not a goal of this work to establish whether or not the degradation leads to the mineralization of the organic compounds, neither the electrolysis time for this, but to know the capacity of the system to produce a usable amount of hydrogen gas as a consequence of the oxidation. In this case, it was found that in a system of only 250 mL of model solution and 20 ppm of MR, for instance, it was possible to produce up to 120 mL of hydrogen gas in 60 min with a faradaic efficiency greater than 80% and with a 100% of MR discoloration.

These results clearly reveal that the electrochemical oxidation of organic compounds, traditionally used as a wastewater treatment, could serve as a coupled process to produce hydrogen gas without high costs as achieved at the typical water electrolysis. Although it is certainly not an electrolyzer, this electrochemical PEM type cell can act as a complementary system of hydrogen production without the energy expenditure being greater than the one invested in the water treatment. Therefore, it is important to recognize that the solution to the energy problem will not come from the development of single systems, capable of covering both aspects of demand and sustainability, but of the specific contribution of integrated devices. Hybrid devices those, in addition to being efficient, are compatible with the environment.

## Conflicts of interest

There are no conflicts to declare.

## Supplementary Material
